# 10. Impact of Infectious Disease Consultation and Theoretical Management Bundle in Patients with Candidemia

**DOI:** 10.1093/ofid/ofab466.010

**Published:** 2021-12-04

**Authors:** ifueko J Adeghe, Dima Kabbani, Tanis C Dingle, Justin Chen

**Affiliations:** 1 University of Alberta, Edmonton, AB, Canada; 2 University of Alberta, Alberta Precision Labs, Edmonton, Alberta, Canada

## Abstract

**Background:**

Candidemia is associated with significant morbidity and mortality. The impact of infectious diseases consultation (IDC) on clinical outcomes in patients with candidemia is not well established. We evaluated the impact of IDC and a management bundle on clinical outcomes in patients with candidemia.

**Methods:**

A retrospective chart review of adult (age ≥ 18 years) patients with at least 1 blood culture growing *Candida* species identified at Alberta Precision Laboratories between December 1, 2019 to November 30, 2020 and hospitalized at the University of Alberta Hospital, Edmonton, Canada were included. Patients who died within 48 hours and those who left against medical advice within 24 hours of initial positive blood culture result were excluded. Demographics, management, and outcome data were collected. A complete management bundle was defined as having all the following elements performed: IDC, repeat blood cultures, empiric echinocandin therapy, ophthalmology consult, and echocardiogram.

**Results:**

Thirty-one patients were included for study; mean age was 56 ± 17 years and 65% were male. 14 (45%) cases were admitted under critical care, 7 (23%) surgery, and 10 (32%) medicine. 3/17 (18%) required intensive care unit admission following candidemia diagnosis. *Candida albicans* was identified in more than half the cases. The primary source was intra-abdominal in 12 (39%), central-line associated in 8 (26%), and urinary in 6 (19%). IDC occurred in 27 cases (87%), echocardiogram in 22 (71%), ophthalmology consult in 10 (32%), and follow-up blood cultures in 30 (97%). 20 (65%) patients received empiric echinocandin. Of the remainder who received empiric fluconazole, 4 (36%) grew non *albicans Candida* species.

Higher in-hospital mortality was observed in cases without IDC than those with IDC (4/4, 100% vs 8/27, 29.6%, p=0.016) and in those that did not have a complete bundle (12/25, 48% vs 0/6, p=0.059). However, IDC was not associated with the receipt of individual bundle components nor the complete bundle (p=NS).

**Conclusion:**

In patients with candidemia, lower in-hospital mortality was observed in patients who received IDC. Larger studies are required to confirm our findings and assess whether the implementation of a candidemia management bundle is beneficial.

**Disclosures:**

**Dima Kabbani, MD**, **AVIR Pharma** (Grant/Research Support, Other Financial or Material Support, Speaker)**Edesa Biotech** (Scientific Research Study Investigator)**Merck** (Scientific Research Study Investigator)

